# Osteogenic Programming of Human Mesenchymal Stem Cells with Highly Efficient Intracellular Delivery of RUNX2

**DOI:** 10.1002/sctm.17-0137

**Published:** 2017-10-31

**Authors:** Lalitha Thiagarajan, Hosam Al‐Deen M. Abu‐Awwad, James E. Dixon

**Affiliations:** ^1^ Wolfson Centre for Stem Cells, Tissue Engineering and Modelling (STEM), Centre of Biomolecular Sciences, School of Pharmacy University of Nottingham Nottingham United Kingdom

**Keywords:** Intracellular transduction, Glycosaminoglycan‐binding enhanced transduction, Cell‐penetrating peptide, Osteogenesis, RUNX2

## Abstract

Mesenchymal stem cells (MSCs) are being exploited in regenerative medicine due to their tri‐lineage differentiation and immunomodulation activity. Currently, there are two major challenges when directing the differentiation of MSCs for therapeutic applications. First, chemical and growth factor strategies to direct osteogenesis in vivo lack specificity for targeted delivery with desired effects. Second, MSC differentiation by gene therapy is difficult as transfection with existing approaches is clinically impractical (viral transfection) or have low efficacy (lipid‐mediated transfection). These challenges can be avoided by directly delivering nonvirally derived recombinant protein transcription factors with the glycosaminoglycan‐binding enhanced transduction (GET) delivery system (P21 and 8R peptides). We used the osteogenic master regulator, RUNX2 as a programming factor due to its stage‐specific role in osteochondral differentiation pathways. Herein, we engineered GET‐fusion proteins and compared sequential osteogenic changes in MSCs, induced by exposure to GET fusion proteins or conventional stimulation methods (dexamethasone and Bone morphogenetic protein 2). By assessing loss of stem cell‐surface markers, upregulation of osteogenic genes and matrix mineralization, we demonstrate that GET‐RUNX2 efficiently transduces MSCs and triggers osteogenesis by enhancing target gene expression directly. The high transduction efficiency of GET system holds great promise for stem cell therapies by allowing reproducible transcriptional control in stem cells, potentially bypassing problems observed with high‐concentration growth‐factor or pleiotropic steroid therapies. Stem Cells Translational Medicine *2017;6:2146–2159*

**Significance Statement**

Many regenerative medicine approaches employ the use of mesenchymal stem cells (MSCs) as they can be obtained directly from the patient from a number of tissues, can be expanded in culture, and have been shown to have positive clinical outcomes in a number of trials. These cells are multipotent meaning they have the ability to become different tissue‐type cells (fat, bone, cartilage) with a predisposition to convert into specific tissue types (differentiate) depending on the source tissue from which they were first isolated. Methods to control or change this predisposition will be key to exploiting them to repair tissue in cell therapies. This article a method to program the gene expression of MSCs to differentiate them efficiently into bone cells. Importantly, this technique can overcome the predisposition to become alternatives (such as cartilage) directly at the level of gene expression. This technology is based upon delivering a recombinant transcription factor protein (RUNX2) which does not genetically modify cells unlike gene therapy. This can now be exploited for programming MSCs when developing strategies for repairing bone trauma and disorders.

## Introduction

New regenerative cellular therapies for bone diseases and trauma often involve the use of patient‐derived or heterologous adult stem cells. Mesenchymal stem cells (MSCs), which are relatively easy to harvest for transplantation, expand and differentiate into multilineages in vitro, and are biocompatible with scaffolds 1. Despite their promise in clinical applications, there are various challenges to consider before MSCs can be used for therapies. Biochemical and molecular interactions in specific MSC niches are highly complex and difficult to reengineer using in vitro cell culture and in tissue regeneration strategies 2, 3. Therefore, understanding and using the molecular factors involved in MSC differentiation for therapeutic applications will be essential for enhancing the success of adult stem cells in therapy.

Currently, predominant strategies to achieve directed differentiation employ the use of chemical induction or exogenous growth factors or hormones, all of which may elicit nonspecific, pleiotropic effects on untargeted cells 4. Ideally, to regenerate bone, osteogenic signaling pathways would be specifically activated using programming factors that perform distinctive roles and that do not affect other related pathways. Therefore, engineering of a targeted regulatory molecule, with higher degree of control over differentiation and an effective, nontoxic delivery system for clinically relevant cell types such as stem cells would significantly improve bone regenerative medicine. One such group of specific regulatory molecules are transcription factors (TFs), which are excellent modifiers of cell fate, play very distinct, stage‐specific roles in differentiation pathway but are very difficult to deliver intracellularly in a biologically active form.

Efficient delivery of these proteins can be achieved by using glycosaminoglycan‐binding enhanced transduction (GET) peptides, which are multidomain peptides comprising a GAG‐binding peptide (to promote cell interaction) and a cell‐penetrating peptide (CPP) for high efficiency membrane transduction 5. We have previously demonstrated that GET can be used to effectively deliver recombinant skeletal muscle TF, MYOD in HEK293T cells to induce zonal myogenesis in a three‐dimensional gradients 6. Here, we wanted to efficiently program human MSCs (hMSCs) using the GET system. Among the various osteogenic TFs, RUNX2 (also called Core‐binding factor alpha, CBFa1) is the most essential for osteoblast commitment, differentiation, matrix production, and mineralization during bone formation 7. RUNX2 regulates downstream genes that determine the osteoblast phenotype and controls the expression of osteogenic marker genes such as *ALP (Alkaline phosphatase), OPN (Osteopontin), OSX (Osterix), COL1A1* (*type‐I collagen), BSP (Bone sialoprotein)*, and *OCN (Osteocalcin)* 2, 3, 8, 9 in response to physiological signals 10. RUNX2 binds to osteoblast‐specific cis‐acting elements (OSE2), which are present in the promoter regions of several osteoblast‐specific genes. Therefore, an appropriate dosage of active RUNX2 is crucial for normal bone development. Interestingly however, in differentiated osteoblasts, RUNX2 needs to be suppressed in order to form mature bone 9, 11. Therefore, RUNX2 activity is explicitly required to trigger the initial osteogenic gene regulatory network and direct the bone developmental program.

Reprogramming or programming factors can be delivered as DNA, RNA, or proteins to manipulate MSC differentiation. However, hMSCs are difficult to transfect; both viral‐ and lipid‐mediated transfection systems have been proven inapplicable for therapies due to virus‐based safety risks and low efficiency in vivo, respectively. Since we have developed the GET delivery method for TFs to efficiently transduce cells, we hypothesized that its use for osteogenic TF delivery could considerably impact hMSC differentiation and drive osteogenic programming. We have used this system to study the effects of osteogenic TF, RUNX2 on osteogenic and chondrogenic differentiation of hMSCs under neutral and pro‐osteo or ‐chondral culture conditions. Our study demonstrates that GET‐RUNX2 could be used to directly trigger the osteogenic gene regulatory network without other osteogenic stimuli and prevent chondrogenesis. As a result, an efficient and specific method of osteogenic induction was developed, removing the need to use pleiotropic compounds (such as dexamethasone), or growth‐factors (such as BMP‐2) which may trigger unwanted off‐target cellular responses.

## Materials and Methods

### Expression and Purification of Recombinant Proteins

8R, P21, RUNX2, and RUNT cDNAs were synthesized de novo (Eurofins MWG Operon, Ebersberg, Germany). We cloned cDNAs into the pGEX6‐P1 expression vector (Novagen Watford, U.K.) and expressed proteins in BL21 (DE3) pLysS *E. coli* (Novagen, Watford, U.K.) as previously described 5. Briefly, exponentially growing LB cultures were induced using 1 mM IPTG for 24 hours at 25°C and sonicated in 1× STE extraction buffer (50 mM Tris, pH 7.5, 150 mM NaCl, 1 mM EDTA containing 1 mM DTT, 0.2 mg/ml lysozyme, and 1× protease inhibitor cocktail). Insoluble protein was retrieved using the Rapid GST inclusion body solubilization and renaturation kit (AKR‐110; Cell Biolabs, Inc., San Diego, CA). GST‐tags were removed by PreScission Protease cleavage (GE healthcare, Amersham, U.K.) in 1× cleavage buffer (50 mM Tris‐HCI pH 7.0, 150 mM NaCl, 1 mM EDTA, and 1 mM DTT). Protein was purified, and the buffer was exchanged to phosphate‐buffered saline (PBS) using Bio‐Spin P6 spin columns (Bio‐Rad, Watford, U.K.). We determined protein concentration using Bradford assay 12. Standards and samples were analyzed using the TECAN infinite 200 PRO multimode reader (Reading, U.K.). Aliquots were stored at −80°C until use.

### Cell Culture

Human mesenchymal stem cells (hMSCs) from two different donors (20 and 21 years; both male; Lonza, Slough, U.K.) were maintained in hMSC growth medium (Lonza, Slough, U.K.) in 5% (vol/vol) CO_2_ humidified incubator at 37°C. hMSCs were subcultured at 80% confluence preventing spontaneous differentiation and contact inhibition of growth. hMSCs were used between passage 4 and 6 for all experiments. All data shown represent three experiments with triplicate samples, unless otherwise stated.

### GET‐Fusion Protein Delivery Assay

To visualize delivery, P21‐RUNX2‐8R was tagged with Fluorescein isothiocyanate (FITC) using NHS (*N*‐hydroxy‐succinimidyl‐ester)‐Fluorescein as per manufacturer's protocol (Thermo Scientific, Paisley, U.K.) at a 1:50 protein: label molar ratio and purified/buffer exchanged to PBS using Bio‐Spin P6 spin columns (Bio‐Rad, Watford, U.K.). A total of 35,000 cells per well (in 24‐well plates) were seeded, incubated for 6 hours for attachment, and transduced overnight with P21‐RUNX2‐8R‐FITC or P21‐mRFP‐8R in growth media. After transduction, cells were washed with PBS, trypsinized with Trypsin‐EDTA (Lonza, Slough, U.K.), and fixed in 4% (wt/vol) PFA (Sigma, Irvine, U.K.) for flow cytometry.

### Osteogenesis Assay

Dulbecco's modified Eagle medium (F12 media; Life technologies, Paisley, U.K.) supplemented with 10% (vol/vol) fetal bovine serum, 2 mM l‐glutamine, 100 units/ml penicillin, and 100 µg/ml streptomycin (Sigma, Irvine, U.K.) was used as the basal media for osteogenic and chondrogenic media. A total of 8,000 cells per well were seeded in a 24‐well plate and cultured for 4 weeks depending on the experiment with appropriate media. Furthermore, 50 μg/ml l‐ascorbic acid 2‐phosphate sesquimagnesium salt hydrate (Sigma, Irvine, U.K.) and 10 mM β‐glycerophosphate disodium salt pentahydrate (Acros Organics, Paisley, U.K.) were added to the basal media for osteo‐permissive medium. To make osteo‐inductive media, 10 or 100 nM dexamethasone (Sigma, Irvine, U.K.) was added to the osteo‐permissive medium. Cells were cultured for 3–4 weeks for complete osteogenesis. For effective osteogenic induction, P21‐RUNX2‐8R (30 μg/ml) was delivered overnight in osteo‐permissive media two times per week during the first week, and the cells were cultured in osteo‐inductive (10 nM dexamethasone) for three more weeks. This delivery strategy was used to induce osteogenesis in hMSCs for further experiments. Medium was changed every other day.

### Chondrogenesis Assay

To determine the chondrogenic potential of hMSCs transduced with P21‐RUNX2‐8R, cells were cultured in two ways; high‐density monolayer on a 24‐well plate or aggregate culture on a U‐bottomed 96‐well plate. A total of 100,000 cells per well (in 96 well round bottom plate) or 600,000 cells per well (in 24 well plate) were seeded and transduced with 30 μg/ml of P21‐RUNX2‐8R overnight in growth media. After transduction, medium was changed to defined chondrogenic medium according to the standard chondrogenesis culture method mentioned by Tew et al. and Penick et al. 11, 13. This defined medium contains basal media supplemented with 10 ng/ml recombinant human TGF‐β1 (Peprotech, London, U.K.), 100 nM dexamethasone (Sigma, Irvine, U.K.), 50 μg/ml l‐ascorbic acid 2‐phosphate sesquimagnesium salt hydrate (Sigma, Irvine, U.K.), 1 mM sodium pyruvate (Life Technologies, Paisley, U.K.), 40 μg/ml l‐proline, and 1× ITS + 1 (Insulin, Transferrin, Selenium, Linoleic acid and bovine serum albumin, Sigma, Irvine, U.K.). The cells were cultured in chondrogenic media for 2 weeks by changing the media every day, and the cultures were taken for further analysis.

### Flow Cytometry

For flow cytometry, cells were trypsinized (unless otherwise stated), fixed in 4% (wt/vol) Paraformaldehyde (PFA), resuspended in PBS (pH 7.5), and analyzed on a MoFlo Astrios (Beckman, Wycombe, U.K.). Flow cytometer using a 488 nm laser (50,000 cells; gated on live cells by forward/side scatter; for P21‐mRFP‐8R transduced cells) or FC 500 (Beckman, Wycombe, U.K.) flow cytometer using 488 laser (30,000 cells for P21‐RUNX2‐8R‐FITC transduced cells; 10,000 cells for immunostained cells; gated on live cells by forward/side scatter). The median relative fluorescence unit was used for statistical analyses after subtracting the background from un‐labeled/transduced cells.

### Microscopy

For bright‐field microscopy of stained cells, the wells were imaged after staining with phase‐contrast inverted microscope Eclipse TS100 (Nikon, Kingston, U.K.). For fluorescent‐labeled and immunostained cells, a Leica DM IRB fluorescence microscope (Leica, Milton Keynes, U.K.) was used to image the cells. For confocal images, P21‐RUNX2‐8R‐FITC transduced cells were fixed, counterstained with Hoechst 33258 (Sigma, Irvine, U.K.), and viewed in Zeiss LSM880 confocal laser scanning microscope.

### Trypan Blue Exclusion Assay

Trypan blue (Fisher Scientific, Loughborough, U.K.) was added to 10 μl of cell suspension in a ratio of 1:1, mixed gently, and then counted using the improved Neubauer hemocytometer (Scientific Laboratory supplies, Nottingham, U.K.).

### Luciferase Reporter Assay

Cells were transfected with firefly *luciferase* reporters (kindly gifted by Dr. Haijun Zhang, Indiana University) mOG2‐Luc or 6XOSE2‐Luc along with the internal control, *Renilla* luciferase reporter pRL‐TK as previously described 14. hMSCs were transduced with the GET‐fusion proteins before, after, or before and after transfection. As a positive control to compare the promoter activity, we transfected hMSCs with pSIN‐RUNX2 plasmid DNA (1 μg, as described in Dixon et al.) 15 using Lipofectamine 2000 (Invitrogen, Paisley, U.K.) and analyzed the luciferase activity. Cells were harvested at different time points, and relative luciferase activities were measured using dual luciferase assay kit (Promega, Southampton, U.K.).

### ALP Assays

After exposure to osteogenic medium for 1 week, cells were washed with PBS and fixed with citrate‐acetone‐formaldehyde fixative and washed again three times with PBS. Extracellular ALP activity was examined histochemically using Naphthol AS‐BI alkaline solution as per manufacturer's protocol (Sigma, Irvine, U.K.). After ALP staining, the samples were washed with PBS and imaged.

### Alizarin Red S Staining

After 28 days, osteogenic cultures were washed three times with PBS and fixed with 4% (wt/vol) PFA and washed thrice with deionized water. Mineralized matrices were stained with 2% (wt/vol) alizarin red solution and quantified using an earlier protocol 16. Briefly, the stained wells were washed three times with PBS, and 200 μl of 10% (vol/vol) acetic acid (Sigma, Irvine, U.K.) was added to each well (24 well plate) and incubated for 30 minutes in a shaker to elute the stain. The eluted stain was heated to 85°C for 10 minutes, cooled, and neutralized with 10% ammonium hydroxide (Sigma, Irvine, U.K.) read at 405 nm using a spectrophotometer. Fold increase in the absorbance value was calculated by comparing with un‐induced cells in osteo‐permissive medium.

### Osteocalcin Immunostaining

After 14 days, osteogenic cultures were rinsed with PBS, fixed in 4% (wt/vol) PFA (Sigma, Irvine, U.K.) in deionized water for 20 minutes, stained with antibody osteocalcin (OCN) (Millipore; 1:200), detected with secondary antibodies conjugated to FITC (Abcam, Cambridge, U.K.), and viewed using fluorescence microscopy. To quantify the OCN positive cells, after 14 days, the cells were trypsinized and stained with antibody against osteocalcin and analyzed by flow cytometry.

### Dimethyl Methylene Blue Assay

To determine the glycosaminoglycan content in chondrogenic cultures, dimethyl methylene blue (DMMB) assay was performed as described by Barbosa et al. 17. Briefly, 21 mg of 1,9‐dimethyl‐methylene blue dissolved in 5 ml of absolute ethanol with 2.0 g of sodium formate and stir thoroughly in 800 ml of distilled water. Concentrated formic acid was titrated into the dye solution to adjust the pH to the desired level (pH 3.0 for the pellet cultures) and made to a final volume of 1,000 ml. The cells were washed after 3 weeks of chondrogenic culture and then lysed with proteinase K (50 μg/ml proteinase K in 100 mM K_2_HPO_4_, pH 8). DMMB solution (1,000 μl) was added to 50 μl of cell lysate or medium supernatant, vortexed vigorously for 30 minutes, and absorbance was measured at 656 nm in a microplate reader. Standard curve was produced using different concentrations of chondroitin‐4‐sulfate (C‐4‐S) to be compared with the samples.

### Alcian Blue Staining

Chondrogenesis of aggregate micromasses of hMSCs were assessed using Alcian Blue staining 18. Chondrogenic cultures were stained for sulfated GAGs with Alcian blue 8GX (Sigma, Irvine, U.K.) solution (1% wt/vol Alcian blue in 0.2 M acetate buffer with 0.06 M MgCl_2_) overnight at room temperature, washed thrice with PBS and images under a dissection microscope.

### Gene Expression Analysis

Total RNA was extracted from hMSCs using RNAeasy kit (Qiagen, Manchester, U.K.) for osteogenic cultures and using TRIzol Reagent (Invitrogen Paisley, U.K.) for chondrogenic cultures according to the manufacturer's instructions. RNA samples were treated with DNase I (Invitrogen), quantified using a NanoDrop 1000 spectrophotometer (ThermoFisher Paisley, U.K.). Osteogenic genes (*ALP–Hs01029144_m1; RUNX2–Hs00231692_m; OSX/SP7–Hs00541729_m1; OCN (BGLAP)–Hs01587814_g1; OPN–Hs00959010_m1*) and chondrogenic genes (*ACAN–Hs00153936_m1* and *SOX9–Hs00165814_m1*) were determined relative to *ACTB* according to the TaqMan gene expression assay protocol (Applied Biosystems/Life Technologies, Paisley, U.K.). TaqMan primers and probes were from Applied Biosystems (Paisley, U.K.). All TaqMan PCR reactions were performed in duplicate with three biological repeats.

### MSC Marker Analysis

MSC marker analysis was done on hMSCs after exposure to different osteogenic conditions by staining them using Stemflow hMSC Analysis Kit (Beckman, Wycombe, U.K.) as per manufacturer's protocol. Mouse anti‐human monoclonal antibodies CD90 FITC, CD73 APC, and CD105 PerCP‐Cy5.5 were used for positive identification of hMSCs. The stained cells were immediately harvested and analyzed on the flow cytometer.

### Statistical Analysis

Statistical comparisons were carried out using GraphPad Prism. The statistical significance was determined using the Holm sidak method for viability and intracellular delivery experiments, one‐way ANOVA for alizarins red S quantification, one‐way ANOVA with Dunnett's post hoc test for MSC marker flow cytometry, two‐way ANOVA for reporter assay, and osteogenic gene expression experiments. Results were considered significant at *p* < .05.

## Results

### Efficient Delivery of RUNX2 in hMSCs Using GET‐Peptides

**Figure 1:**
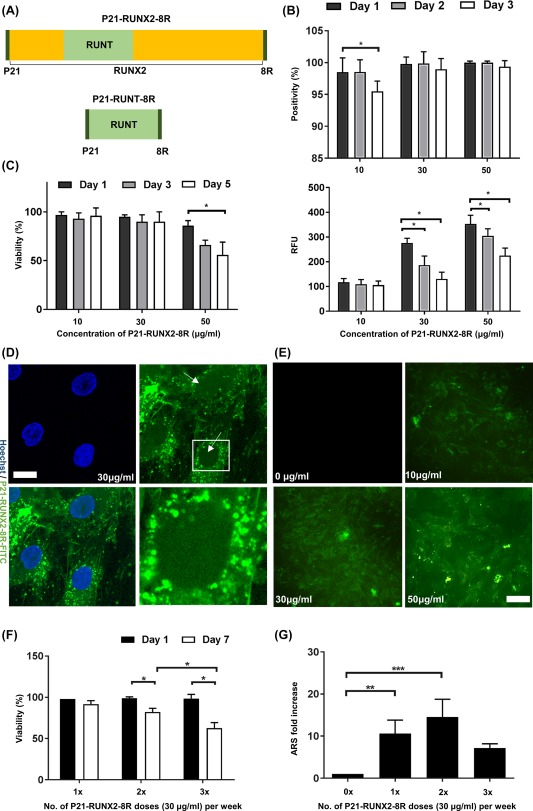
Efficient delivery and dosing of P21‐RUNX2‐8R to initiate osteogenesis in human mesenchymal stem cells (hMSCs). **(A):** Design of the osteogenic constructs. P21‐RUNX2‐8R is a RUNX2 transcription factor with an N‐terminal fusion of P21 and a C‐terminal fusion of 8R. P21‐RUNT‐8R contains only the DNA binding domain, RUNT, sandwiched between P21 and 8R. **(B):** To assess the delivery of the fusion peptide, the proteins were labeled with FITC and delivered at different concentrations overnight. Flow cytometry analysis of percentage positivity and relative fluorescence unit of hMSCs treated with different concentrations of P21‐RUNX2‐8R‐FITC (10, 30, 50 μg/ml) overnight. Statistical significance was determined using the Holm‐Sidak method, *α* = 0.05;*, *p* < .05. **(C):** Viability of hMSCs measured using trypan blue at day 1 and 7 after overnight treatment with 30 μg/ml of P21‐RUNX2‐8R. Thirty μg/ml of P21‐RUNX2‐8R was used as the optimal concentration on further experiments. Statistical significance determined using the Holm‐Sidak method, with α = .05; *, *p* < .05. **(D):** Confocal images of hMSCs treated with 30 μg/ml P21‐RUNX2‐8R‐FITC overnight and counter stained with Hoechst (nuclei stain) at ×40 magnification. Significant amounts of P21‐RUNX2‐8R‐FITC are colocalized with the Hoechst stained cell nucleus. Most P21‐RUNX2‐8R‐FITC is localized in small endosomal vesicles present in perinuclear region in the cytoplasm (hatched area). Scale bar is 10 μm. **(E):** Fluorescence microscopy images of hMSCs treated with 10, 30, and 50 μg/ml P21‐RUNX2‐8R‐FITC overnight. As the concentration increases, fluorescence intensity of P21‐RUNX2‐8R‐FITC inside hMSCs increases. Scale bar is 50 μm. **(F):** For the first week, hMSCs were treated overnight with P21‐RUNX2‐8R (30 μg/ml) every other night (3×), every 3 days (2×), or treated only once (1×). Viability of hMSCs measured using trypan blue at day 1 and 7 after overnight treatment with 30 μg/ml of P21‐RUNX2‐8R once, twice and thrice per week. Statistical significance determined using the Holm‐Sidak method, with α = 0.05; *, *p* < .05. **(G):** Treated hMSCs were cultured in osteo‐permissive medium for 3 weeks, stained with alizarin red S (ARS) for matrix mineralization and quantified using a microplate reader. Thirty μg/ml of P21‐RUNX2‐8R treated twice during the first week was identified as the optimal dose, and the same conditions were used in the subsequent assays. Statistical significance was determined using one way ANOVA, with *α* = 0.05;*, *p* < .05; **, *p* < .005; ***, *p* < .001. Error bars indicate standard deviation (SD). hMSCs from two different donors were used for this study.

GET peptides are potent in promoting macropinocytosis and can enhance intracellular transduction of conjugated molecules by orders of magnitude over simple CPPs such as octarginine (8R) or HIV‐derived TAT in many cells types 5. Importantly, we have previously demonstrated that fusion of GET peptides to TFs as recombinant proteins enhance intracellular delivery over CPPs with retention of TF transcriptional regulatory activity 5. To build on our previous studies, we characterized the transduction of a fluorescent reporter (mRFP) into primary hMSCs. Recombinant proteins are not significantly transduced into hMSCs without supplementation with GET peptides, P21‐8R (Supporting Information Fig. S1a). We focused on the osteogenic TF, RUNX2 which is vital for triggering the osteogenic gene regulatory network and has concentration‐dependent, stage‐specific roles in osteochondral development and differentiation. We fused a bacterially optimized human *RUNX2* cDNA directly to the P21 and 8R peptides (5′ and 3′, respectively), to generate a P21‐RUNX2‐8R expression construct for bacterial expression. The DNA binding domain of RUNX2, termed RUNT, was also generated as an inhibitory molecule, as its overexpression has been previously shown to block RUNX2‐mediated transcriptional activation and prevent osteogenesis through competitive binding 19, 20, 21. Both GET‐tagged and untagged variants of RUNX2 were expressed and purified as recombinant proteins in *Escherichia coli*. To visualize intracellular delivery of these TFs, we labeled the purified recombinant protein with NHS‐FITC (Fig. 1A). In order to visualize P21‐RUNX2‐8R‐FITC localization, we used confocal microscopy (Fig. 1D) and observed significant amounts of P21‐RUNX2‐8R‐FITC colocalized with the Hoechst stained cell nucleus. However, most protein is localized in small endosomal vesicles present in perinuclear region in the cytoplasm. Controlling for labeling efficiency, we observed a dose‐dependent increase in the uptake of all recombinant GET fusion proteins in hMSCs. Untagged proteins showed no significant uptake even at dosages 10‐fold (100 μg/ml) of that used for GET‐fusions. Both GET‐fused RUNX2 and RUNT showed a decrease in the viability of cells at the highest dosages used or most frequent treatment regimens (>30 μg/ml or >2 dosages of 20 μg/ml per week) (Fig. 1C, 1D) which is not observed with delivery of the mRFP reporter (Fig. S1b). This is unsurprising as although RUNX2 is a key TF regulating osteogenesis, it has been shown to trigger cell apoptosis with significant overexpression 22. Furthermore, the dosages used could achieve an effective supraphysiological level of the TF. As the dosing was a simple exposure to media containing the GET‐fusion proteins, we could rapidly tailor the dosage to that which did not affect cell viability and less likely to drive off‐target effects of gene expression (<30 μg/ml).

### Optimal Dose of P21‐RUNX2‐8R Significantly Increases Mineralization

For hMSC osteogenic differentiation in vitro, ascorbic acid and β‐glycerophosphate (AB) are essential for final stages of bone nodule formation by promoting collagen matrix production and providing inorganic phosphate for mineralization, respectively, 4. In vivo these supplements would be available to the developing bone naturally within the body. In order to test the effect of P21‐RUNX2‐8R and P21‐RUNT‐8R on complete/terminal bone differentiation programming, this media (AB) condition termed “osteo‐permissive” was used and compared with that lacking these additives. Importantly, the osteo‐permissive environment does not induce or trigger initiation of any osteogenesis (by gene expression assessment or immunological assessment) but augments the final differentiated bone phenotype. hMSCs were exposed to P21‐RUNX2‐8R for 7 days in osteo‐permissive medium by transducing (overnight exposure) the cells once, twice, or thrice per week, and any pro‐osteogenic effect assessed. We optimized these conditions to deliver an effective dose but not to affect the viability of hMSCs by overdosing at supraphysiological levels. This is important as overexpression of RUNX2 causes severe disorders in transgenic mice at supraphysiological activities 2. P21‐RUNX‐8R indeed triggered osteogenic differentiation which was evident from the significant calcium mineralization, quantified using alizarin Red S staining 28 days postdelivery. RUNX2 without GET peptides did not initiate osteogenesis, and there was no evident mineralization. Furthermore, GET‐tagged RUNX2 significantly promoted osteocalcin expression (immunocytochemistry) which was undetectable in controls or untagged RUNX2 cultures (Supporting Information Fig. S2a). We assessed the gene expression programming in treated cultures, with early, mid, and late responsive genes (previously shown to mark osteogenesis) significantly activated, unlike control or untagged comparators (Supporting Information Fig. S2b). We observed the maximum amount of mineralization with least effect on viability by delivering P21‐RUNX2‐8R twice per week (30 μg/ml dose) during the first week, followed by no further delivery and culture in osteo‐permissive medium up to 28 days (Fig. 1E, 1F). We conducted other optimization experiments with continuous lower dosages (10 and 20 μg/ml) over the 28 day experiment. Lower continuous dosages produced significant but lower levels of mineralization. Continued dosing significantly inhibited the final mineralized phenotype to the point that no mineralization was evident if a continuous high‐dose of GET‐tagged RUNX2 was used. We therefore optimized a high‐dose, short‐term treatment regime to effectively induce osteogenesis in hMSCs. It is known that *RUNX2* levels and relative levels to *SOX9* are important for key fate decisions in osteogenic differentiation, programmed at the transcriptional level 23, 24. These results are therefore intriguing as they demonstrate that the initial priming of osteogenesis and not later stages require RUNX2 activity, and furthermore high RUNX2 activity over the entire developmental program can be inhibitory, especially when cells are terminally differentiating to produce a mineralized phenotype. It is interesting to note that Loebel et al. demonstrated effective osteogenesis induction through *SOX9* downregulation leading to increased *RUNX2/SOX9* ratio 23.

### P21‐RUNX2‐8R Promotes Rapid Loss of the Multipotent MSC Phenotype

**Figure 2:**
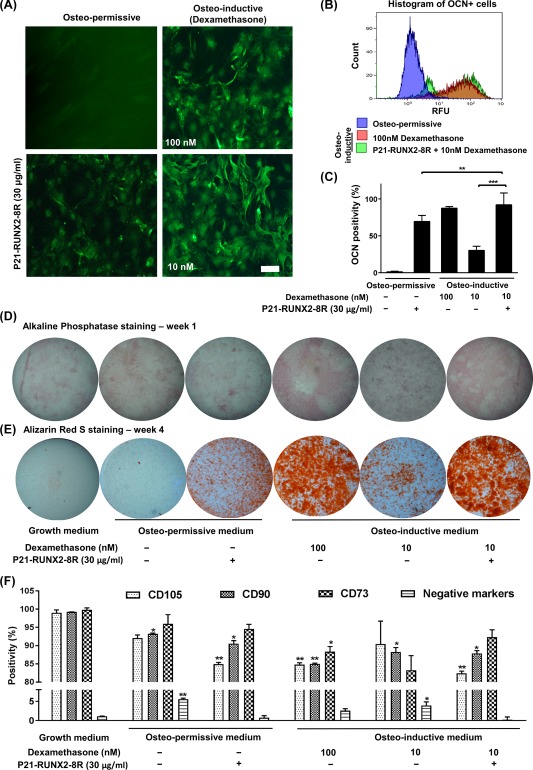
P21‐RUNX2‐8R programs human mesenchymal stem cells (hMSCs) toward osteogenesis. **(A):** Representative fluorescent microscopic images of hMSCs cultured in osteo‐permissive medium or in osteo‐inductive medium (100 nM or 10 nM dexamethasone) with or without 30 μg/ml of P21‐RUNX2‐8R (treated overnight, twice per week) and stained for OCN after 3 weeks. Scale bar is 20 μm. **(B, C):** Flow cytometry was performed after OCN staining to measure percentage positivity. Two distinct population of differentially stained OCN^+^ cells can be observed in P21‐RUNX2‐8R treated hMSCs in the histogram. Statistical significance was determined using multiple *t* test in comparison to hMSCs cultured in osteo‐permissive medium, with α = 0.05; *, *p* ≤ .05; **, *p* ≤ .005; ***, *p* ≤ .001. RFU is relative fluorescence units. **(D):** Representative images of hMSC cultures after 1 week under different treatments stained for alkaline phosphatase. Images of the wells were taken in a dissection microscope at ×1.5 magnification. **(E):** Representative images of matrix mineralization assessment by alizarin red S staining on hMSC cultures with different treatment after 4 weeks. Images of the wells were taken in a dissection microscope at ×1.5 magnification. **(F):** P21‐RUNX2‐8R modifies the expression of bone marrow specific MSC markers toward bone differentiation. hMSCs were cultured in different conditions for 2 weeks, stained for mesenchymal stem markers, and analyzed by flow cytometry. Statistical significance was determined using one‐way ANOVA with Dunnett's post hoc test in comparison to the undifferentiated nontreated control hMSCs, with α = 0.05; *, *p* ≤ .05; **, *p* ≤ .005. Error bars indicate SD. hMSCs from two different donors were used for this study. Abbreviations: OCN, osteocalcin; RFU, relative fluorescence unit.

We aimed to confirm that P21‐RUNX2‐8R directly induced the commitment of hMSCs toward osteogenic differentiation with a concomitant loss of hMSC phenotype. Using flow cytometric analysis, we performed MSC phenotyping by measuring surface markers (CD73, CD90, CD‐105) as recommended by the International Society of Cellular Therapy (ISCT) 25. We labeled GET‐RUNX‐transduced cells with hMSC markers and assessed the timing of MSC marker downregulation with upregulation of the osteogenic program. We observed an initial decrease of CD105 (0.2‐fold; *p* < .05), CD90 (0.2‐fold *p* < .05), and slight downregulation (0.08 fold; *p* < .05) of CD73 in both P21‐RUNX2‐8R transduced and dexamethasone‐treated osteogenic cells (Fig. 2F) after 12–14 days of culture compared with noninduced controls. Reduction in CD105 and CD90 antigens represents cellular commitment to nonmyocardial lineages 26, and maturation toward osteoblast‐like cells 27, respectively. Interestingly, CD73 antigen retention suggests osteochondral pathway differentiation 28. Previously it has been shown that MSC populations with higher CD79 and CD39 positivity exhibit higher expression of SOX9 and RUNX2 29, 30, 31. Our data agree with this study and indicate heightened osteochondral differentiation potential triggered through P21‐RUNX2‐8R exposure 29, 30, 31, 32.

To directly confirm rapid generation of reciprocal osteogenic marker upregulation during exposure, we assessed transduced cells for the early osteogenic marker, OSTEOCALCIN (OCN). OCN expression confirms full osteogenic commitment before progression toward bone nodule formation 33. We observed a strong expression of OCN in P21‐RUNX2‐8R transduced cells early (14 days) postexposure with the highest expression observed 21 days post‐treatment (Fig. 2A, 2C). Interestingly, we routinely detected two cellular populations of OCN: high and low positive cells (Fig. 2B). This variation in OCN expression is likely to be generated from the heterogeneous potency of starting population of hMSCs leading to asynchronous induction toward osteogenesis 34, 35, 36. Importantly, due to the efficient initiation of osteogenesis by P21‐RUNX2‐8R, leading to almost complete programming of the culture, any heterogeneity in the starting MSC population can be effectively overridden, inducing osteogenesis in cells likely to have a propensity for differentiation into other lineages (such as adipogenic and chondrogenic lineages) 37.

### Transcriptional Activity of Transduced P21‐RUNX2‐8R Is Higher than Exogenously Overexpressed RUNX2

**Figure 3:**
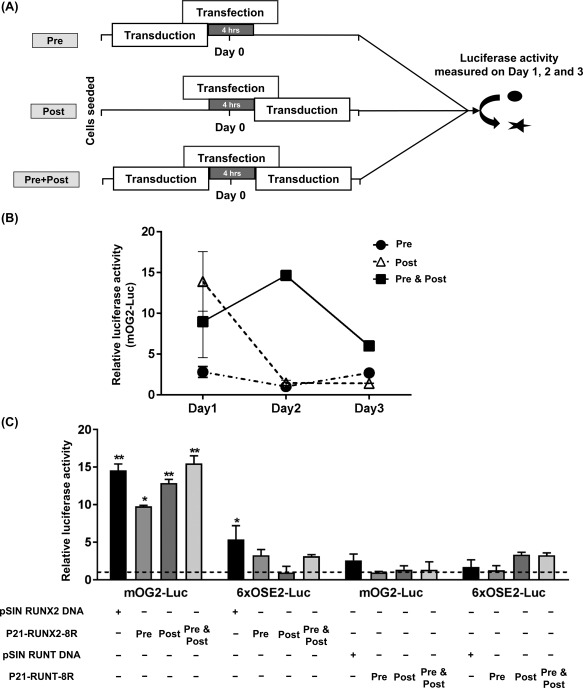
P21‐RUNX2‐8R significantly activates osteocalcin promoter. **(A):** Schematics of the set‐up to assess the transcriptional activity of P21‐RUNX2‐8R and P21‐RUNT‐8R using dual luciferase assay by transfecting human mesenchymal stem cells (hMSCs) using Lipofectamine 2000 with a luciferase‐based osteocalcin promoter reporter (mOG2‐Luc or 6XOSE2‐Luc), *Renilla* luciferase promoter with or without plasmids (pSIN RUNX2/RUNT DNA—as positive control for the assay) as indicated. During transduction, the cells were treated with the proteins (30 μg/ml) overnight. The cells were transduced with the protein before (Pre) or after (Post) or before and after (Pre & Post) the reporter transfection. **(B):** Luciferase activity was determined after day 1, 2, 3 on Pre, Post and Pre & Post conditions on hMSCs. Statistical analysis was performed using two‐way ANOVA, α = 0.05. **(C):** Both the reporters (mOG2‐Luc and 6XOSE) were tested for luciferase activity on all three conditions (Pre, Post, Pre & Post) by transducing with GET‐fusion proteins, P21‐RUNX2‐8R or P21‐RUNT‐8R. Relative luciferase activity was determined 28–32 hours after reporter transfection. Base level luciferase expression of nontransfected and nontransduced cells are represented with dotted lines and compared with other treatments for statistical analysis using multiple *t* test, with α = 0.05; *, *p* ≤ .05; **, *p* ≤ .005. Error bars indicate SD. hMSCs from two different donors were used for this study.

In order to confirm transcriptional activity of P21‐RUNX2‐8R in the most direct assay possible, we examined immediate transcriptional activation of RUNX2‐responsive luciferase reporters containing the transcriptional responsive sequence from the *OSTEOCALCIN* (*OCN*) promoter. To optimize the dual luciferase assay for maximum transcriptional induction, we transduced test proteins at different time points in relation to reporter transfection (before, after, or both) (Fig. 3A, 3B). We observed that dosing cells pre‐, post‐ and pre‐, and post‐transfection produced significantly different transcriptional induction levels (using the mOG2‐Luc reporter). We determined that predosing and postdosing generated the highest induction by P21‐RUNX2‐8R. As positive and negative controls, we used pDNA vectors with strong EF1α‐promoters (pSIN vectors) 15 to drive either *RUNX2* or *RUNT* cDNA expression, respectively. We observed P21‐RUNX2‐8R induces mOG2‐Luc reporter expression to a greater magnitude to pSIN‐RUNX2 plasmid (p)DNA (15.5‐fold vs. 14.3‐fold). A synthetic reporter containing six copies of the OSE2 element from the *OSTEOCALCIN* (*OCN*) promoter (6xOSE2‐Luc) also induced with P21‐RUNX2‐8R delivery or RUNX2 plasmid, but to a lower magnitude than the mOG2‐Luc reporter. Neither plasmid‐expressed RUNT (pSIN‐RUNT) nor P21‐RUNT‐8R transduction had significant effect on the reporters (Fig. 3C) as expected. Importantly, these data demonstrate direct activity immediately after transduction of P21‐RUNX2‐8R. The kinetics of the effect on the transfected luciferase reporters are clearly different to that of coexpressing TFs from plasmids which is as expected; GET‐delivered TFs can, therefore, directly access the cell nucleus and regulate gene expression immediately upon intracellular transduction; unlike DNA vectors which require transcription/translation before produce any activity.

### P21‐RUNX‐8R Triggers Early Lineage Specification but Inhibits Terminal Differentiation

**Figure 4:**
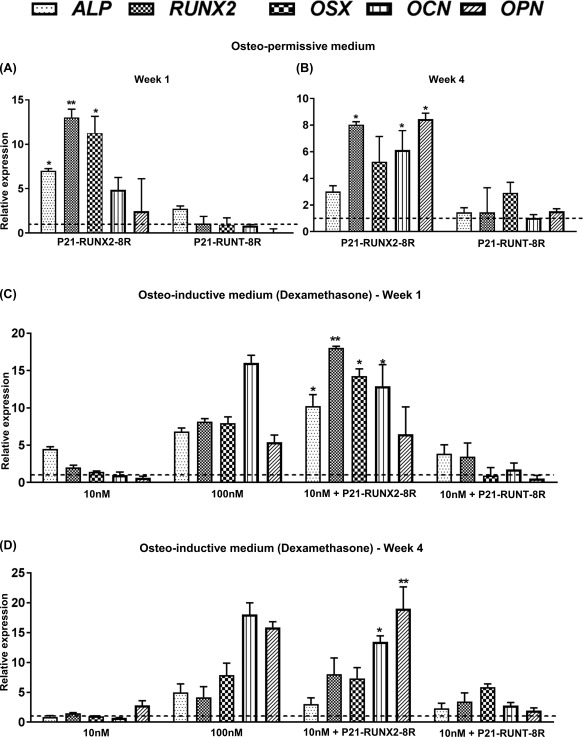
P21‐RUNX2‐8R significantly activates osteogenic genes. Human mesenchymal stem cells (hMSCs) were cultured in osteo‐permissive and osteo‐inductive media with and without protein transduction. The wells were washed and total RNA was extracted after 1 and 3 weeks. After DNAse treatment, gene expression analysis of *ALP, RUNX2, OSX, OCN,* and *OPN* of P21‐RUNX2‐8R or P21‐RUNT‐8R transduced hMSCs on Week 1 **(A, C)** and 4 (**B, D**), cultured in osteo‐permissive (A, B) or osteo‐inductive medium (C, D) was performed. The results were plotted on the graph based on expression fold change to nontransduced hMSCs cultured in osteo‐permissive medium (dotted line). Statistical analysis was performed using two‐way ANOVA in comparison with the nontransduced hMSCs, with α = 0.05; *, *p* ≤ .05; **, *p* ≤ .005. Error bars indicate SD. hMSCs from two different donors were used for this study. Abbreviations: ALP, alkaline phosphatase; OPN, Osteopontin; OSX, Osterix.

Next, we examined the effect of P21‐RUNX2‐8R on early and late marker genes for osteogenesis. P21‐RUNX2‐8R significantly induces endogenous *RUNX2* expression both in osteo‐permissive and osteo‐inductive medium after week 1 (Fig. 4A, 4C). *OSTERIX* (*OSX*), a TF immediately downstream of *RUNX2*, is equally induced post‐transduction (Fig. 4A–4d) confirming the initiation of osteogenic pathways 38. Expression of *Osteopontin* (*OPN*), an important component in bone nodule formation 39, however, was lower in osteo‐permissive medium with P21‐RUNX2‐8R transduction in comparison to dexamethasone‐induced cells. *OSTEOCALCIN (OCN)* (*BGLAP*, bone gamma‐carboxyglutamic acid‐containing protein), a protein secreted by mature osteoblasts, is significantly upregulated during week 4 in P21‐RUNX2‐8R treated cells cultured in osteo‐permissive medium (Fig. 4B, 4D). The expression pattern is comparable to the results obtained through OCN immunostaining and quantification, where addition of 10 nM dexamethasone significantly enhanced OCN expression in P21‐RUNX2‐8R treated cells (Fig. 2A–2C). Considerable change in gene expression profile can be seen between week 1 and week 4 cultures both in osteo‐permissive and osteo‐inductive media between these induction methods. *RUNX2* expression is significantly higher (13.4‐fold over noninduced control; *p* < .01) during the first week than in week 4 (8.2‐fold over noninduced control; *p* < .05) when cultured in osteo‐permissive media using P21‐RUNX2‐8R. This terminal decrease in *RUNX2* expression during osteogenesis was observed and suggested as essential for osteoblast function in RUNX2‐dependent osteogenesis studies by Liu et al. 40. Addition of P21‐RUNT‐8R did not induce any significant downregulation in *RUNX2* and *OSX* although reduction in *OPN* expression was observed (0.9‐fold decrease compared with nontransduced control) (Fig. 4A). This suggests that P21‐RUNT‐8R has an inhibitory effect on osteogenesis. Addition of subthreshold dose of dexamethasone (10 nM), instead of 100 nM (used for full osteogenic induction), efficiently enhanced *OPN* and *OCN* expression in P21‐RUNX2‐8R transduced cells after 4 weeks of culture (Fig. 4D; Supporting Information Table S1). It is interesting to note that P21‐RUNX2‐8R delivered with 10 nM dexamethasone doubled *RUNX2* and *OSX* expression in comparison to 100 nM dexamethasone in week 1 cultures (Fig. 4A). Addition of 10 nM of dexamethasone without P21‐RUNX2‐8R did not have major effect on any osteogenic marker genes both in short‐term and long‐term cultures. The same can be confirmed for matrix mineralization of cultures under these conditions stained using alizarin red S (Fig. 2E). We speculate that this might be due to the extremely low concentration of dexamethasone which is not adequate to activate the necessary gene regulatory networks to initiate osteogenesis as observed previously by Walsh et al. 41.

Alkaline phosphatase (ALP) present in bone‐related cells is a tissue nonspecific hydrolytic enzyme which is active in multiple other locations such as liver and kidney. We performed gene expression analysis of *ALP* (tissue nonspecific gene variant) of our cultures and found pronounced expression during early osteogenesis with P21‐RUNX2‐8R, both in osteo‐permissive and osteo‐inductive conditions (Fig. 4A, 4C). However, ALP staining of these cultures did not show significant variation among the treatments (Fig. 2D). We assessed cultures for ALP activity at day 7 since we found the staining of transduced cells increased with every dose of P21‐RUNX2‐8R with maximum expression between day 5 and day 8. Since ALP can be found in various tissue sources without bone formation and the ALP expression during osteogenesis varies significantly, this marker is not ideal to confirm genuine osteogenic induction 12, 42.

To further examine how P21‐RUNX‐8R can augment osteogenic programming in comparison to other bone differentiation strategies, we examined the effect of transduction on BMP‐2‐stimulation of hMSCs. Both BMP‐2 (growth factor) and dexamethasone (steroid) stimulation can be enhanced with co‐stimulation of cells with P21‐RUNX2‐8R (Supporting Information Fig. S3a), but this does not have the pleiotropic effects seen on other gene‐regulatory programs. BMP‐2 without dexamethasone induction did not induce osteogene expression when added at the start of osteogenic culture. Also, the common use of dexamethasone in osteogenic as well as chondrogenic induction media for MSCs reinforces the pleiotropic effects of such steroids. The expression of all the genes tested were less (<0.5‐fold decrease in *RUNX2* and *OSX* expression; 0.95‐fold decrease in *OPN* expression, *p* > .05) in comparison to cells in osteo‐permissive media alone (Supporting Information Fig. S3a). These data suggest that P21‐RUNX2‐8R could also represent a tool to create a more “sensitive” osteo‐permissive transcriptional condition for other bone differentiation strategies.

### P21‐RUNX‐8R Does Not Trigger Osteogenesis Pleiotropically

In order to assess if P21‐RUNX2‐8R could enhance bone differentiation in other cell types, we tested its effects on cell types which cannot naturally undergo osteogenic differentiation. This is important as an understanding of the possible pleiotropic effect that P21‐RUNX2‐8R might have on viability and osteogene expression in other cell types could affect how it could be applied clinically. No significant increase or decrease in osteogene expression was observed in P21‐RUNX2‐8R transduced endothelial (HUVECs; 0.02‐fold increase compared with nontransduced cells, *p* > .05) (Supporting Information Fig. S4b) and a major decrease in osteogenic gene expression was observed in fibroblasts (HUES7 hESC‐derived fibroblasts; >0.6‐fold decrease in *RUNX2* and *OPN* expression compared with nontransduced cells, *p* > .05) (Supporting Information Fig. S4c). Interestingly, small but significant increases in *OSX* expression (fourfold, *p* > .05 in HUVECs and twofold increase, *p* > .05 in HUES7 hESC‐derived fibroblasts), and a minor decrease in viability was observed with dexamethasone (100 nM) in both cell types (Supporting Information Fig. S4). Overall, these data suggest that P21‐RUNX2‐8R does not possess pleiotropic activity, such as that demonstrated here and in the literature for dexamethasone‐ or BMP‐2‐strategies 43, 44, 45, 46.

### P21‐RUNX2‐8R Induced Osteogenesis Inhibits Chondrogenesis

In order to understand the effect of P21‐RUNX2‐8R on the crosstalk between osteogenesis and chondrogenesis differentiation pathways, we transduced hMSCs at a chondrogenesis‐conductive seeding density, cultured in osteogenic or chondrogenic media and analyzed the gene expression profile of osteogenic and chondrogenic genes. Two‐dimensional (low and high seeding density) and three‐dimensional culture (round bottom aggregate culture) systems were initially assessed (Supporting Information Figs. S5 and S6) and used to test the transduction efficiency of GET and chondrogenesis potential of these cultures with transduction. Consequently, we compared cell culture that induces chondrogenesis (aggregates formation) in the presence and in the absence of osteogenic and chondrogenic media and assessed if P21‐RUNX2‐8R can override these cues to promote an osteogenic program. Protein transduction was carried out prior to aggregate formation to ensure high transduction efficiency as aggregates showed <50% transduction positivity (Supporting Information Fig. S5b).

Our extensive expression analyses demonstrated *SOX9* expression was significantly reduced in transduced cells in both high‐density monolayer (0.53‐fold decrease compared with nontransduced control) and aggregate culture (0.43‐fold decrease compared with nontransduced control) with chondrogenic medium. It is interesting to note that addition of P21‐RUNX2‐8R suppresses *SOX9* in both culture conditions.

**Figure 5:**
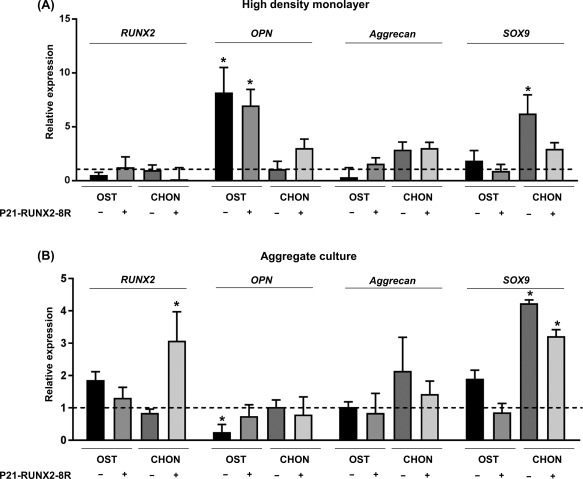
P21‐RUNX2‐8R significantly inhibits chondrogenic genes under osteogenic conditions. Human mesenchymal stem cells (hMSCs) were cultured in osteo‐inductive media (with 100 nM Dexamethasone) with or without protein transduction under two different seeding conditions mentioned below. The cells were washed and total RNA was extracted after 2 weeks. After DNAse treatment, gene expression analysis of osteogenic genes (*RUNX2* and *OPN*) and chondrogenic genes (*Aggrecan* and *SOX9*) of P21‐RUNX2‐8R transduced and nontransduced hMSCs was performed. The cells were cultured as a high‐density monolayer on flat bottom plate **(A)** or as aggregate masses **(B)** on a round bottom plate in osteo‐inductive medium with 10 nM Dexamethasone (OST) or chondrogenic (CHON) medium. The results were portrayed in a graph based on expression fold change to un‐transduced hMSCs cultured in basal media (dotted line). Statistical analysis was performed using two‐way ANOVA, with *α* = 0.05; *, *p* ≤ .05. Error bars indicate SD. hMSCs from two different donors were used for this study. Abbreviation: CHON, chondrogenic; OPN, Osteopontin.

In aggregate culture, P21‐RUNX2‐8R effectively supresses chondrogenic gene expression, *SOX9* and *ACAN*, and increases *RUNX2* expression in chondrogenic medium after 2 weeks, suggesting that P21‐RUNX2‐8R meticulously steers the signaling pathway toward osteogenesis even under chondrogenic conditions. This might also suggest hypertrophy as higher expression of *RUNX2* at terminal stages of culture could mean hypertrophy or intramembranous ossification 15. Overall, chondrogenic genes (*SOX9* and *ACAN*) were relatively less expressed in osteogenic medium compared with chondrogenic medium under both culture conditions (Fig. 5A, 5B).

*SOX9* and *RUNX2* expression ratio is crucial in determining the shift in equilibrium toward osteogenesis or chondrogenesis 47, 48, 49. In spite of chondrogenic conditions and increased *SOX9* expression, osteogenes were expressed in both culture conditions in the presence of P21‐RUNX2‐8R. *SOX9/RUNX2* ratio, chondrogenesis‐conducive aggregate culture, and osteogenesis inductive P21‐RUNX2‐8R can likely promote osteoclast formation needed for osteochondral differentiation. Similar gene expression profile was observed in the works of Schagemann et al. and Glueck et al., where TGFβ (present in chondrogenesis medium) enhances chondrocyte differentiation, osteoblast maturation, and osteoclast maturation 50, 51.

## Discussion

MSCs are a heterogeneous population of cells, and their lineage inclination, differentiation potential, and functional properties are often determined by their tissue source. Various reports have been published detailing that MSCs from certain tissues favor particular biological characters, for example, bone‐derived MSCs are more prone to osteogenic lineages 35; synovial membrane‐derived MSC prefer chondrogenic lineage 52; amnion‐derived or chorion‐derived MSCs not only have multilineage differential potential but also possess immune suppressive ability 36, 53. Also, isolating and purifying homogeneous population of MSCs are very difficult. Even the surface marker‐based enrichment of MSCs does not produce highly purified population with the same clonogenic and differentiation potential 54, 55. Lack of well‐defined MSC biomarkers, variability in the MSC phenotype, and clonal heterogeneity of MSCs make it essential to understand the molecular signaling biology of their differentiation so as to effectively direct lineage‐specific tissue regeneration 56, 57.

Genetic alteration using viral vectors to program MSCs in a regenerative therapy scenario brings safety concerns involving genetic modification and deviations in cell fate caused by transient changes in transcriptome after DNA delivery 58, 59, 60, 61. Therefore, manipulating MSCs by targeting upstream molecular targets using small molecules will enable safer and effective therapeutic application 34. Direct programming of cells using exogenously delivered TFs can by‐pass growth factor complexity, but these technologies heavily rely on viral delivery or have low efficacy.

TFs are the backbone of regulatory networks in the cell that orchestrate stage‐specific gene expression or repression in order to prompt a biological function 4. Gene expression control can be achieved without altering the genetic sequence by the use of TFs. TFs act as mediators in the regulatory network that governs various cellular functions such as differentiation, proliferation, development, and immunomodulation. Developmental and cell‐type specific genetic transcriptional regulators are activated or repressed by the integration of multiple signaling molecules which is highly complex to control. Fundamental behavior such as cell fate, growth, and death are programmed through the interpretation of these signals and by the activity of transcription regulators. These transcription regulators are used in programming, reprogramming, or transdifferentiation of cells in vitro such as PU.1 and C/EBP α and β that transforms fibroblasts into macrophage‐like cells 62, or a combination of GATA4, TBX5, NKX2.5, and BAF60c (GTNB) converting human embryonic stem cells and fibroblasts into cardiomyocytes 15. These TFs can also be delivered as proteins which can be readily taken up by the cells thereby alleviating the need for DNA/RNA transfection or viral delivery. Here we have built upon our previous demonstration that MYOD coupled with intracellular delivery peptides (GET peptides) enhance myotube formation in HUES7 embryonic stem cells 5.

Use of intracellular delivery peptides to transport TFs inside the cells for reprogramming opens a wide range of application in regenerative therapy. In order to test this delivery peptide plus TF concept on biological functions, we focussed on differentiating hMSCs into osteocytes. Conventional methods of osteogenesis from hMSCs for regenerative therapies involve the use of growth factors or chemicals. Bone morphogenetic protein 2 (BMP‐2), a member of transforming growth factor (TGF) superfamily, is involved in both bone and cartilage development. Although BMP‐2 has been in orthopaedic use for bone injuries in clinical application 45, the capacity of BMP‐2 to initiate MSC commitment toward osteogenesis over chondrogenesis is not completely understood 4, 63, 64, 65. Various clinical reports have highlighted operative site edema complications in craniomaxillofacial and spinal applications using recombinant human BMPs 43, 44, 45, 46. Dexamethasone is a common component added in the media for osteogenic induction in vitro. It is a corticosteroid clinically used for inflammatory diseases such as arthritis, ulcerative colitis, psoriasis, lupus, and other allergic disorders, and hence it has been cautiously used in bone‐related therapies in vivo due to its possible pleiotropic effects 4, 64, 66, 67. To overcome these problems, RUNX2, the key master regulator of bone formation was used as the osteogenesis initiator in hMSCs. Here, we have created a transducible‐version of RUNX2 with GET peptides. With this approach, we could also develop better targeted strategies to re/program stem cells by using loaded or coated microparticles for controlled release of the factors. Presently, we are developing controlled release strategies for GET‐RUNX2 and these will be applied to bone‐repair models in vivo. Our strategy of delivering specific factors for enhancing bone formation also helps in preventing undesirable undirected differentiation or off‐target or systemic side‐effects. Furthermore, as this represents an efficient nonviral delivery system there will be less regulatory aspects to resolve before clinical application.

## Conclusion

We demonstrated that the GET peptides promoted TF delivery thereby directing the differentiation toward a desired lineage. The ability to trigger specific differentiation programs while isolating the effects of competing stimulation at the transcriptional level will allow more precise control of cell programming to direct cellular behavior for many regenerative medicine applications. We believe that our improvement of the long‐established ex vivo osteogenesis methods has the potential to become a patient‐applicable translational technology for cell‐based therapies and regenerative medicine.

## Author Contributions

J.E.D.: conceived and initiated the project; L.T., H.A.M.A.A., and J.E.D: designed and performed experiments; J.E.D supervised the project; L.T. and J.E.D.: manuscript writing. L.T., H.A.M.A.A. J.E.D. Approvel the final manuscript.

## Disclosure of Potential Conflicts of Interest

The authors indicated no potential conflicts of interest.

## Supporting information

Supplementary Figures
