# Supplementary material for: Osteogenic Programming of Human Mesenchymal Stem Cells with Highly Efficient Intracellular Delivery of RUNX2
Source: Stem Cells Transl Med. 2017 Oct 31;6(12):2146–59. doi: 10.1002/sctm.17-0137 (PMC5702512; doi:10.1002/sctm.17-0137)
Supplement: Supplementary file 1 — Supplementary Figures [file SCT3-6-2146-s001.docx]

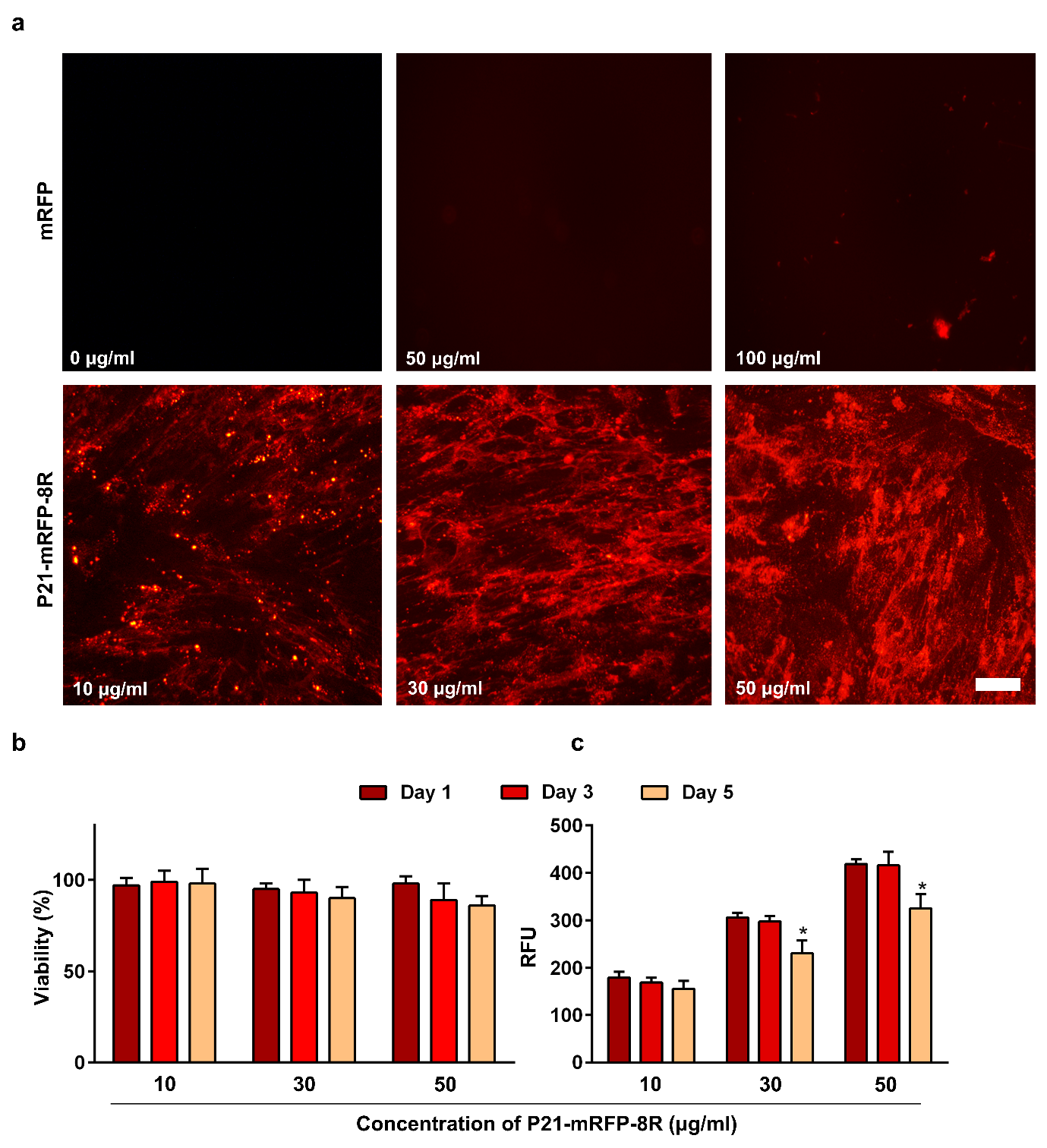


**Supplementary figure 1: P21-8R is essential for protein delivery in hMSCs**

**a**) Representative fluorescent microscope images of hMSCs transduced with mRFP (50 and 100μg/ml) and P21-mRFP-8R (10, 30 and 50 μg/ml) overnight. Scale = 50 μm. **b**) Cells treated with different concentrations of P21-RUNX2-8R for overnight were trypsinised 24 hour after transduction and viability was determined using trypan blue. **c**) After transduction, the cells were harvested for flow cytometry analysis at Day 1, 3 and 5. Statistical significance determined using the Holm-Sidak method, with α=0.05; * p ≤ 0.05. Error bars indicate SD.


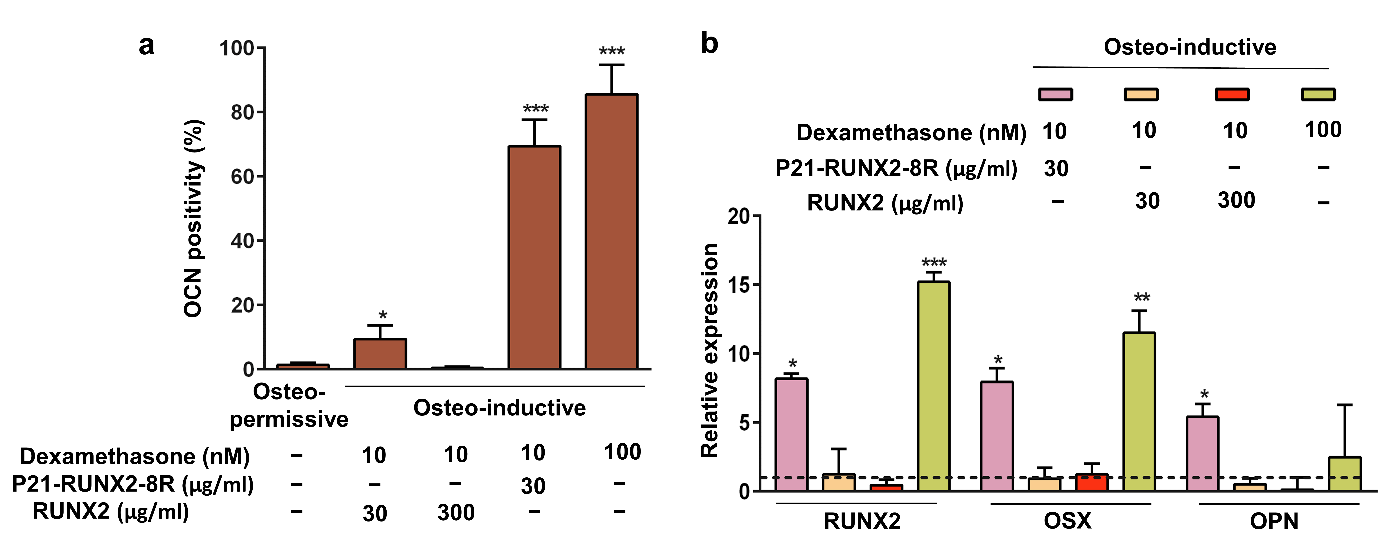


**Supplementary figure 2: P21-8R is essential for osteogenesis initiation by RUNX2 in hMSCs.**

**a**) For the first week, hMSCs were transduced with RUNX2 (30 or 300 μg/ml) or P21-RUNX2-8R (30 μg/ml) overnight twice per week and cultured in osteo-inductive medium. Cells cultured in osteo-permissive medium was used as the negative control. After two weeks, the cells were trypsinized, stained for OCN and analysed by flow cytometry. Statistical significance was determined using multiple t-test in comparison to hMSCs cultured in osteo-permissive medium, α=0.05; * p ≤ 0.05; ** p ≤ 0.005; *** p ≤ 001. **b**) After two weeks of culture, total RNA was extracted and the expression of RUNX2, OSX and OPN were analysed. The results were plotted on the graph based on expression fold change to non-transduced hMSCs cultured in osteo-permissive medium (dotted line). Statistical analysis was performed using two-way ANOVA in comparison with the non-transduced hMSCs, α=0.05; * p ≤ 0.05; ** p ≤ 0.005; *** p ≤ 001. Error bars indicate SD.


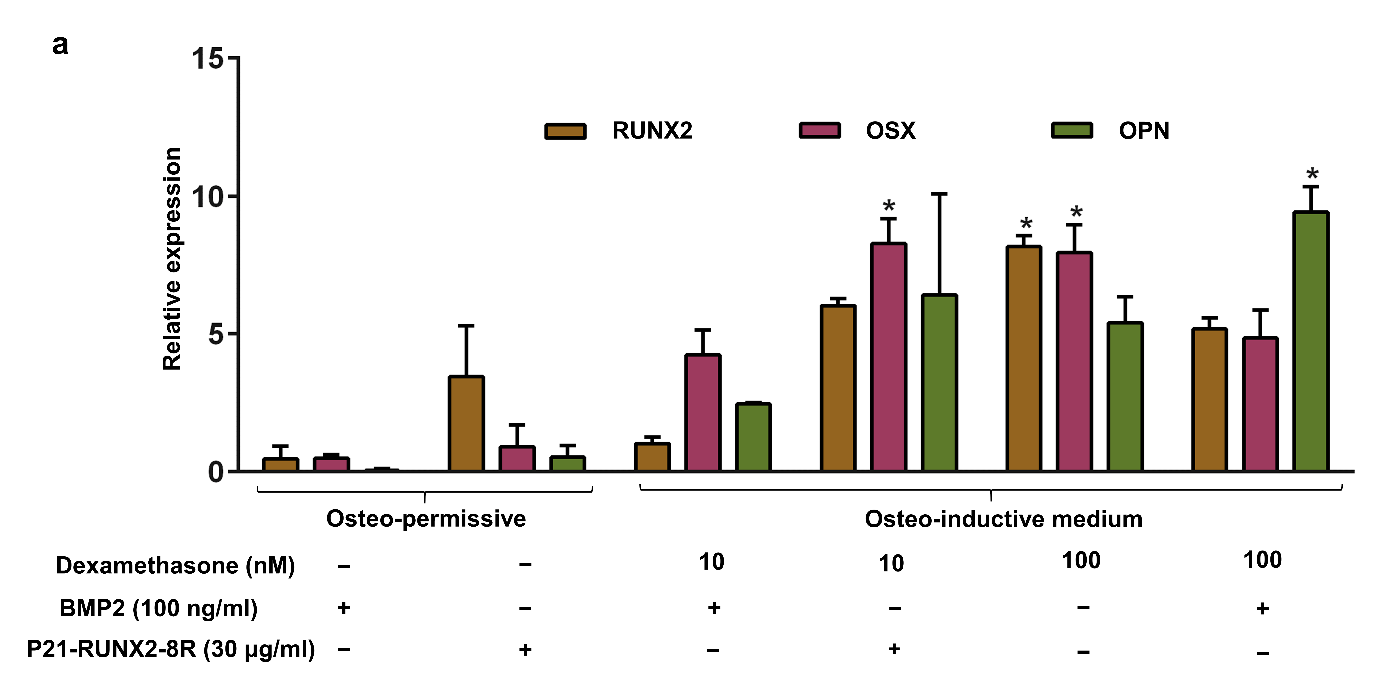


**Supplementary figure 3: P21-RUNX2-8R induction of osteogenesis is similar to BMP2.**

**a**) P21-RUNX2-8R and BMP2 based induction of osteogenesis were compared. For the first week, hMSCs were transduced overnight with P21-RUNX2-8R (30 μg/ml) twice per week, are cultured in osteo-inductive medium. hMSC were treated with BMP2 (100 ng/ml) for two weeks, then cultured in osteoinductive medium. After two weeks, the cells were trypsinised, stained for osteocalcin and analysed by flow cytometry. Statistical analysis was performed using two-way ANOVA in comparison with the non-transduced hMSCs, α=0.05; * p ≤ 0.05. Error bars indicate SD.


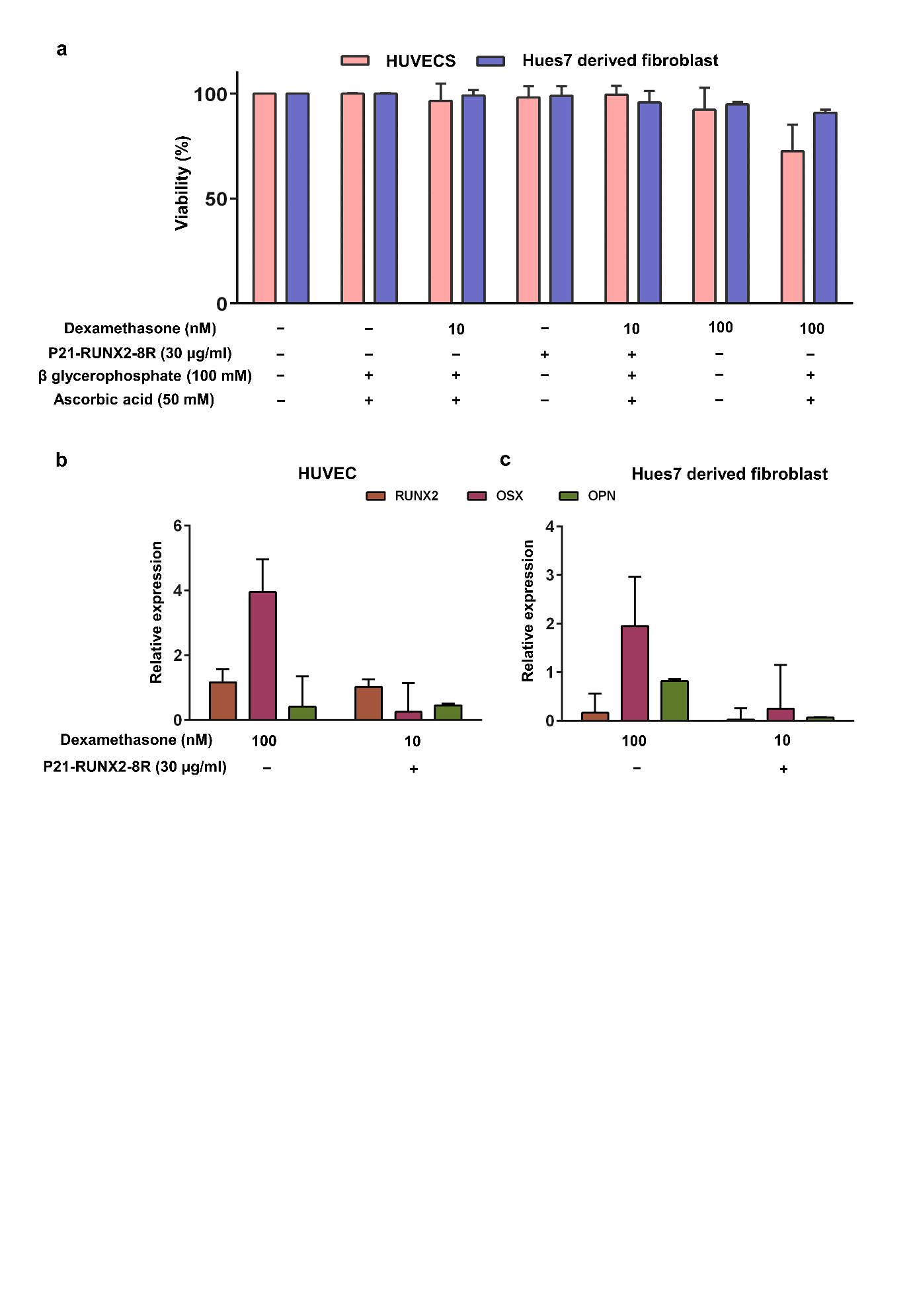


**Supplementary figure 4: P21-RUNX2-8R did not affect viability or initiate differentiation in non-targeted cells.**

**a**) Viability was assessed using trypan blue under different treatments after three days of culture. **b** and **c**) P21-RUNX2-8R did not induce osteogenesis in non-targeted cells. For the first week of culture, HUVECS and HUES7 derived fibroblast were transduced with P21-RUNX2-8R (30 μg/ml) overnight twice per week, After three weeks total RNA was extracted and expression of RUNX2, OSX and OPN was analysed for HUVECS (**a**) and Hues7 derived fibroblast (**b**). Statistical analysis was performed using two-way ANOVA in comparison with the non-transduced hMSCs, α=0.05. Error indicate SD.


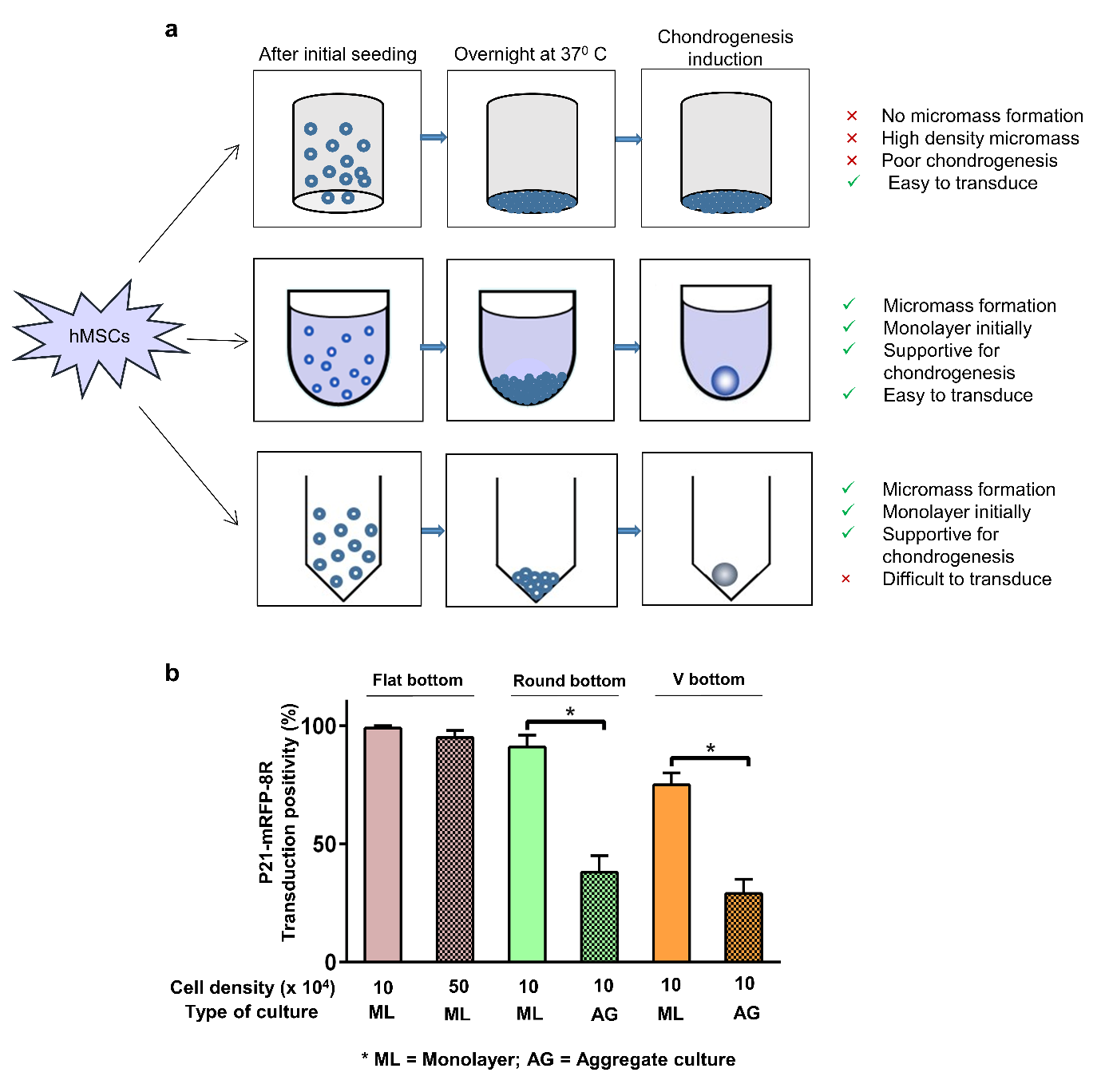


**Supplementary figure 5: GET-peptides are effectively transduced even in high density monolayer.**

**a**) Schematics of the culture conditions assessed to determine a condition that encourages both chondrogenesis and transduction. **b**) P21-mRFP-8R effectively transfects high density monolayer but not aggregated cell masses. hMSCs were seeded at different seeding densities in flat, round and v bottom 96 well plates and are transduced as a monolayer or as aggregate micromasses. Cells were trypsinised 24 hour post transduction and fixed. Transduction efficiency of P21-mRFP-8R was measured using flow cytometry after overnight transduction with the peptide in different culture conditions. Statistical significance was determined using one way ANOVA, α=0.05; * p ≤ 0.05. Error bars indicate SD.


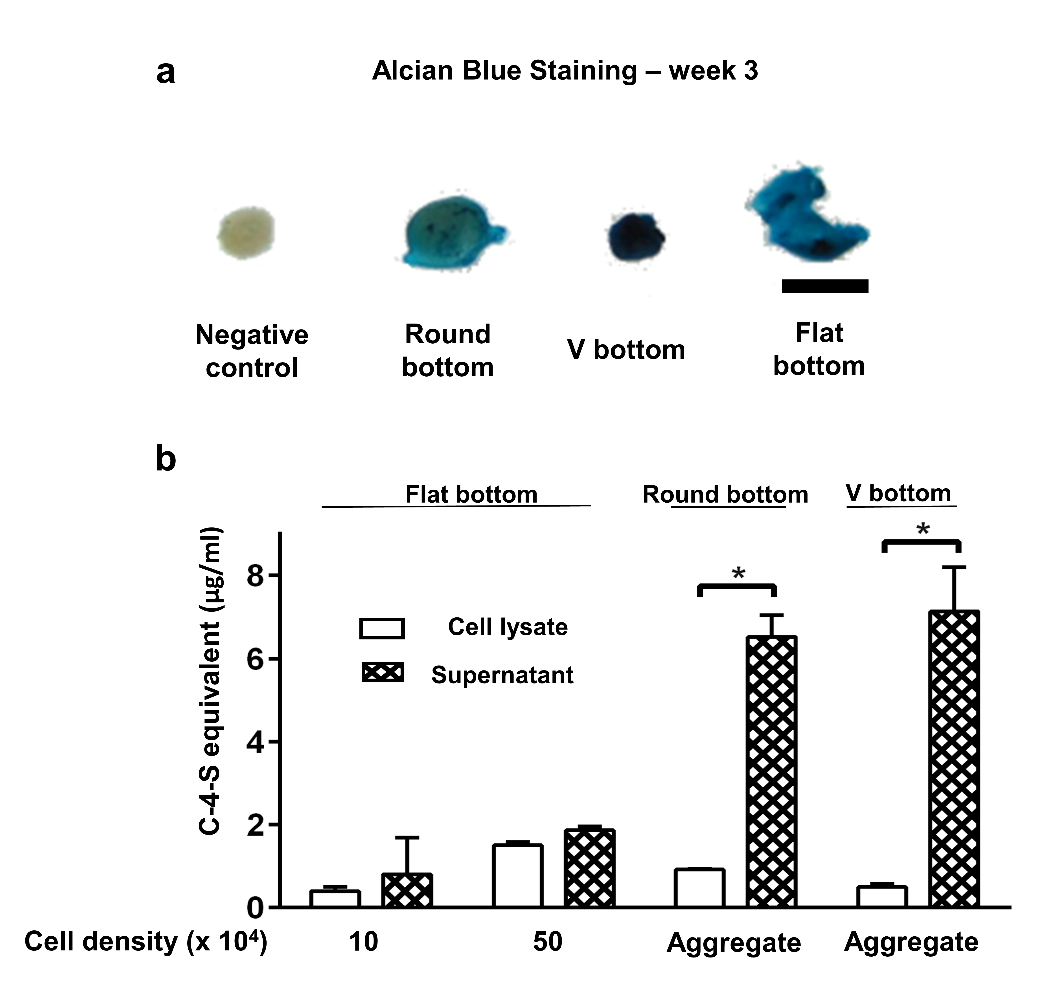


**Supplementary figure 6: Round bottom wells support chondrogenesis and transduction.**

**a**) Alcian blue staining showed GAG distribution on the aggregates. After three weeks of chondrogenic culture, the aggregates were stained with Alcian blue and imaged under dissection microscope. Scale = 2 mm. **b**) Aggregated cell masses produce increased amounts of glycosaminoglycan (GAG) detected by DMMB assay. hMSCs were seeded at above mentioned seeding density in flat, round and v bottom 96 well plates. Cells were cultured in chondrogenic media for 3 weeks and GAG content was quantified in the cell lysates and in the medium. Statistical significance was determined using one way ANOVA, α=0.05; * p ≤ 0.05. Error bars indicate standard deviation (SD).
